# More Research Is Needed on Lifestyle Behaviors That Influence Progression of Parkinson's Disease

**DOI:** 10.3389/fneur.2019.00452

**Published:** 2019-04-30

**Authors:** Nupur Nag, George A. Jelinek

**Affiliations:** Neuroepidemiology Unit, Melbourne School of Population and Global Health, The University of Melbourne, Melbourne, VIC, Australia

**Keywords:** Parkinson's disease, lifestyle behaviors, observational studies as topic, longitudinal studies, multimodal treatment concept

## Abstract

The variability of symptoms in Parkinson's disease (PD) suggests the need for individualized treatment. A key aspect of precision medicine is lifestyle risk factor modification, known to be important in the prevention and management of chronic illness including other neurological diseases. Diet, cognitive training, exercise, and social engagement affect brain health and quality of life, but little is known of the influence of lifestyle on PD progression. Given disease heterogeneity, absence of objective outcome measures, and the confounding effects of medication, investigating lifestyle as a potential therapy in PD is challenging. This article highlights some of these challenges in the design of lifestyle studies in PD, and suggests a more coordinated international effort is required, including ongoing longitudinal observational studies. In combination with pharmaceutical treatments, healthy lifestyle behaviors may slow the progression of PD, empower patients, and reduce disease burden. For optimal care of people with PD, it is important to close this gap in current knowledge and discover whether such associations exist.

## Introduction

Parkinson's disease (PD) is an age-related complex progressive neurodegenerative disorder, with key pathological features being the presence of alpha-synuclein-containing Lewy bodies and a loss of dopaminergic neurons in the substantia nigra (1). Years to decades preceding diagnosis, symptoms can include constipation, sleep behavior disorder, hyposmia, and anxiety (2). At diagnosis, hallmark motor symptoms of bradykinesia, as well as either resting tremor or rigidity, are defining (3).

The spectrum of motor and non-motor symptoms, and their impact on patient quality of life, suggests a need to individualize treatment. Current treatments primarily act to replace or boost existing dopamine, managing mostly motor symptoms. However, their long-term use leads to side effects, and reduced efficacy (4). Treatment of non-motor symptoms, including fatigue and cognitive impairment, is often secondary though they can have a significant impact on daily living (5, 6).

A broader range of therapeutic alternatives is needed to manage symptoms and ideally slow PD progression. The difficulty in therapeutic discovery is partially attributed to limited understanding of PD pathogenesis, assuming similar disease mechanisms across clinically heterogeneous patients, and the absence of biological markers to measure disease progression (7, 8). Nevertheless, as the spectrum of individual symptoms is increasingly being recognized, precision medicine is receiving warranted attention.

**Figure 1:**
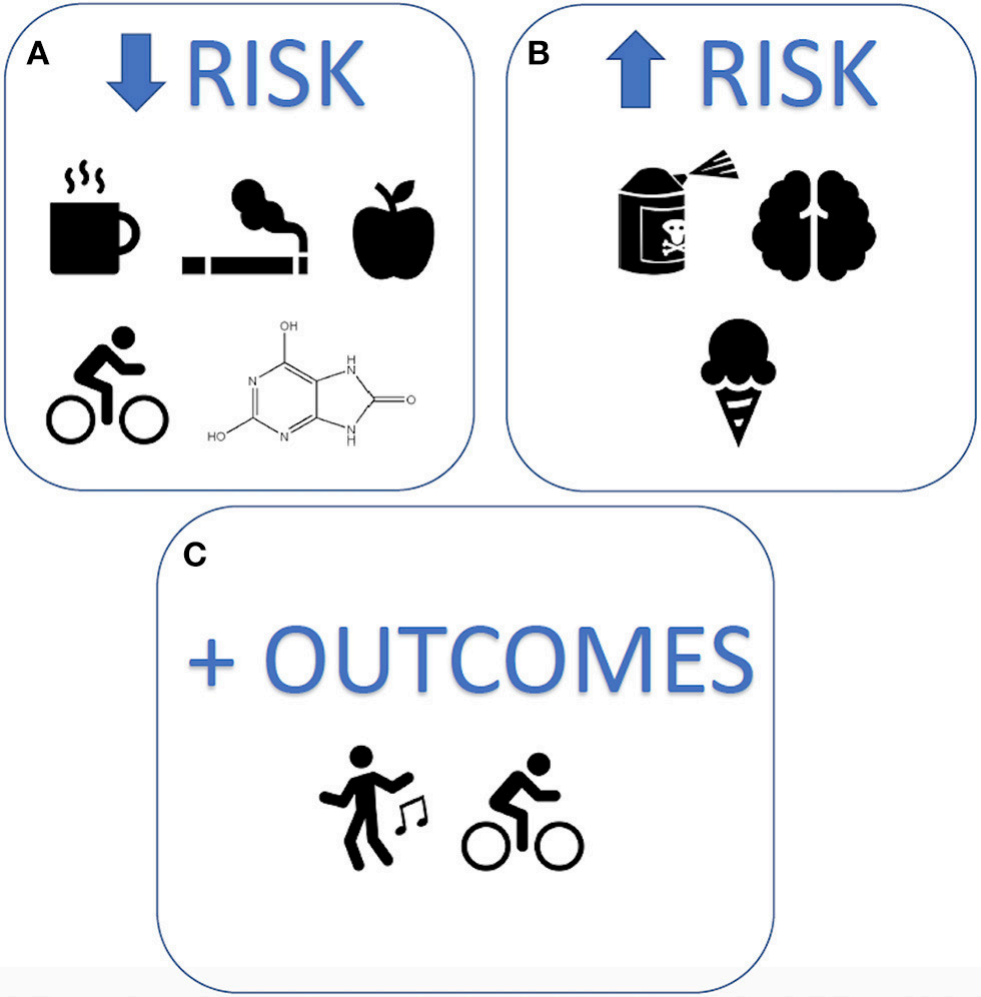
Modifiable lifestyle factors associated with Parkinson's disease risk and outcomes. The strongest lifestyle factors associated with Parkinson's disease, reported to date, include **(A)** reduced risk: caffeine, smoking, uric acid, quality diets, and exercise **(B)** increased risk: exposure to pesticides, head injury, and dairy products, and **(C)** improved outcomes: mind-body exercises and physical activity.

A key aspect of precision medicine is attention to modifiable lifestyle risk factors, including nutrition and exercise, known to be important to neuronal health (9–11), and potentially important in secondary prevention of progression of PD. Several studies have shown associations between modifiable lifestyle factors and PD risk and outcomes (Figure 1). Reduced risk of developing PD is associated with physical activity and perversely with smoking, while increased risk is associated with constipation and anxiety or depression (12). Mind-body practices and endurance exercise can improve PD health outcomes (13, 14), however their long-term effects on neuroprotection or disease-modifying potential in PD remain inconclusive (4, 12, 15). Similarly, despite associations observed between PD risk and urate, dairy, and caffeine, the effects of nutrition on progression remain unclear (15–17). Further research is required to elucidate the long-term effects of lifestyle behaviors on PD management and progression if secondary prevention of PD with lifestyle modification is to be a realistic treatment option.

## Study Designs to Measure Lifestyle Behaviors

Randomized controlled trials (RCTs) are the gold standard to examine therapeutic efficacy of an intervention (18, 19). However, selection bias, randomization, adherence, and short study duration often make RCTs impractical for lifestyle studies. In any event, there is scant information on which lifestyle factors might even be tested in such studies. To discover potential lifestyle exposures that might benefit neuronal health in PD and warrant trialing, unbiased monitoring of a population for lengthy periods is required. Here, registries can provide a valuable tool.

Barriers in establishing population-based registries include recruitment, cost, and data quality. While opt-out enrolment avoids recruitment bias, registries require close to 100% capture of patients with the disease in a given demographic. Extraction of data elements from patient electronic health records can save cost and time, with better data quality. Successful registries require significant collaborative efforts from clinicians and trained staff, to contribute data to a centralized repository. Time-poor clinicians may be reluctant to participate, and issues of data access, ownership, and governance can be additional barriers.

A cost- and time-efficient approach is an embedded trial within an existing database (18, 19). With this approach, a database with high quality data is required. Most existing databases capture predominantly Caucasian participants, recruit from hospitals, have low incident cases of PD, and collect little data on lifestyle behaviors (20). These issues could be lessened by combining comparable multi-center international cohorts and adding lifestyle variables to datasets. The success of combining cohorts necessitates a commitment to collaboration, standardized data definitions, data management and governance, and significant ongoing funding.

Observational cohort studies are less resource intensive than RCTs, and useful for complex study protocols and small patient populations (18). Selection bias and participant drop-out may be addressed through multifactorial recruitment and active engagement methods such as free access to wellbeing classes, and regular communication through newsletters, public seminars, and interactive workshops. Information bias and confounding may be minimized by design and analysis (19). In addition to efficiency, benefits of observational studies include minimal participant effort and adherence issues, as one follows natural behaviors.

**Figure 2:**
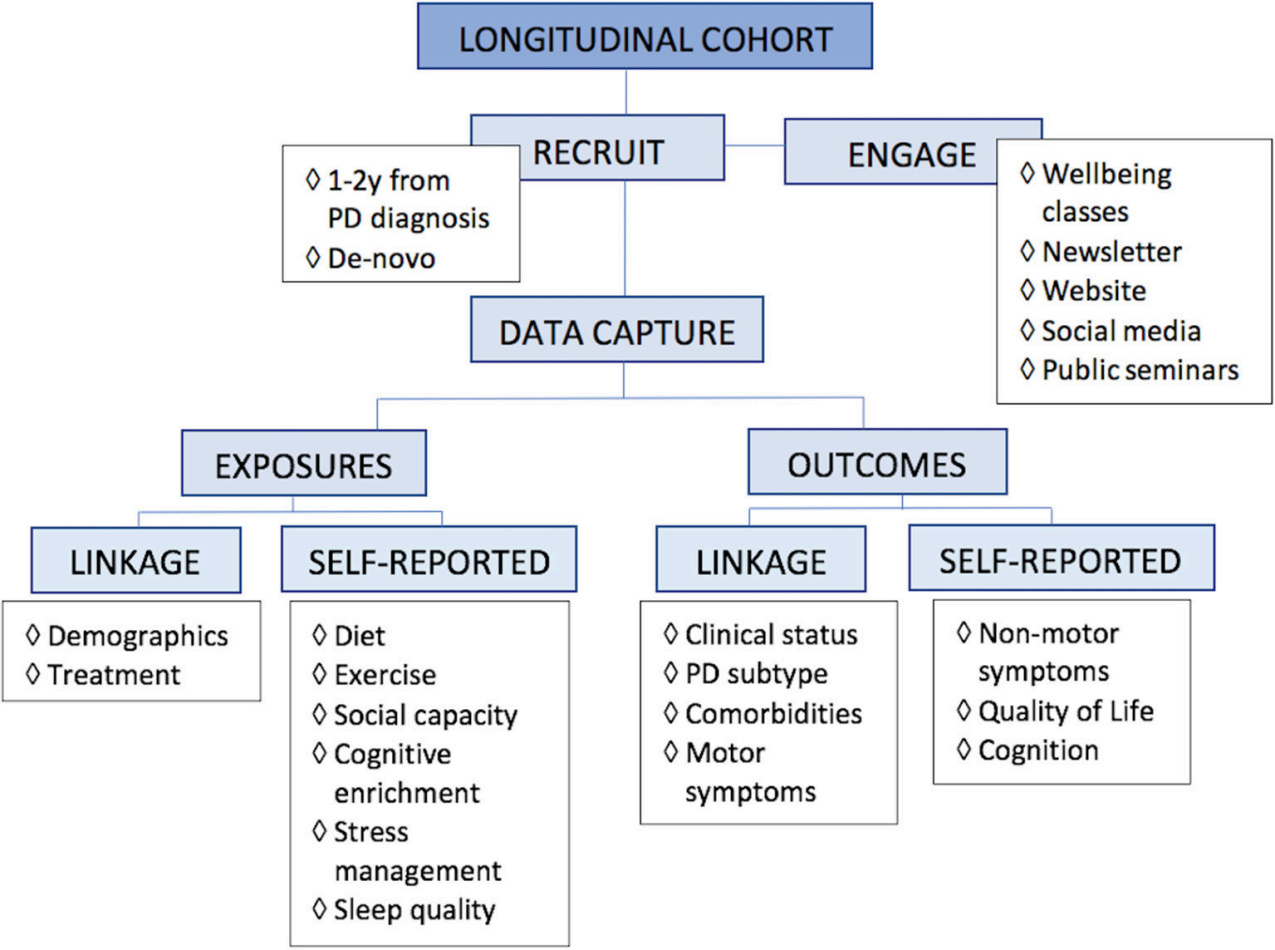
Proposed research design for lifestyle. For research into multimodal lifestyle factors that impact Parkinson's disease, we propose a longitudinal study of an enriched population, capturing data via linkage, and self-reported online surveys.

Given that someone may follow more than one aspect of a healthy lifestyle, observational studies are most practical to evaluate associations of lifestyle and health outcomes. A proposed research design would be a longitudinal cohort study, with inclusion of an enriched PD population, caputuring data via a combination of data linkage to diagnosing and treatment clinics as well as self-reported online surveys (Figure 2). Selecting appropriate data variables to capture requires scientific rationale, with consideration of feasibility, practicality, and cost-effectiveness. The ability of potential recommendations to be seamlessly incorporated into people's everyday lives also needs to have a bearing on data capture.

Registry and observational studies can provide informed decisions for areas of focus for RCTs (18). Ideally, any strong association should be verified with a RCT prior to clinical recommendation. However, where common sense points to beneficial effects of low-risk modifiable behaviors on stress reduction, weight management, and cognitive engagement, health professionals may choose to prioritize patient education to incorporate healthy lifestyle into daily living.

## Limitations of Existing Lifestyle Studies in PD

Several databases capture data on aging community members, as well as people at high risk of or diagnosed with PD. A 2017 study reviewed 44 of 68 identified PD databases around the world, showing that many include few incident cases of PD, little data on lifestyle, and were of limited duration (20). The authors highlight an unaddressed opportunity to combine these databases, thereby increasing research collaboration and knowledge of PD with a larger patient cohort.

Variability of interventions, improper controls, lack of relevant outcomes measures, and recruitment bias, make results of existing studies difficult to interpret or generalize (4). Additionally, there is no distinction or stratification of participants based on PD stage or subtype, which delineate disease symptoms and rate of progression (21, 22).

Questions remain unanswered on minimal dose requirements, distinction of a learnt response, sustainable effects once intervention ceases, as well as the impact of aging, baseline health, and comorbidities. The significant lack of evidence points to the need for an ongoing large-scale database to capture and monitor lifestyle and health outcomes in people with PD.

## Challenges of Lifestyle Observational Studies

Selection bias, confounding, and recruitment are key challenges. Multifactorial recruitment strategies and appropriate analysis can minimize selection bias and confounders, respectively (19). Screening for an enriched cohort may increase recruitment efficiency and the possibility of observing a therapeutic effect. Prodromal cohorts allow identification of PD in its earliest stages, with time to conversion being a measure of disease progression. Algorithms based on a combination of risk factors group participants into high, medium, and low risk of conversion, thereby potentially isolating an enriched, trial-ready population (23). Interventions are likely to have the most effect on this high-risk group as neurodegeneration is less established. Primary limitations are identifying participants with prodromal features, lack of generalizability given a selective PD sub-type, slow conversion of up to 14 years, and distinguishing intervention effects from slow rate of conversion.

Within diagnosed groups, extensive neuronal damage may result in barely perceptible effects of lifestyle changes, and these may only affect non-motor symptoms. *De novo* participants with both short prodromal phase and time from diagnosis are favorable subjects, however misdiagnosis is common in this early phase (24). Most patients will be medicated within 12 months of diagnosis, after which time the effects of interventions are difficult to untangle. Measures of disease progression in diagnosed cohorts may therefore need to include time to pharmaceutical treatment, stable medication dose, motor or cognitive decline, and neuroimaging.

Study duration and participant retention are additional challenges. Lifestyle signals may be modest; therefore, an observational plan needs to be made for at least 5 years to see meaningful progression of the disease. Research funding is typically granted for 2–3 years, limiting potential for such data collection. To encourage retention, researchers should engage with participants by regularly communicating study milestones and other relevant and useful information, as well as promote involvement in events. Creative reminders and motivators to complete surveys with accuracy, to ensure unbiased data collection and analysis, are also important.

## Outcome Measures

Lifestyle interventions are hard to measure precisely and may produce very specific and subtle signal changes. High baseline levels of healthy living are likely to be neuroprotective, thus increasing these levels may produce little change in health outcomes (25). Each intervention component should be measured at baseline and adjusted for effect size. Ideally, this would be measured with a combination of physiological markers and clinical assessments.

The development of markers of PD risk, diagnosis, and progression is a priority. Advances have been made for potential risk and diagnostic markers, including smell and sleep tests, imaging to detect dopamine neurotransmitter, alpha-synuclein, in the peripheral nervous system or cerebrospinal fluid, and gene variants in family members. As yet, no biomarker has however been validated as reliable or replicable for clinical use and none exists to measure disease progression (26). While important to provide insight into potential mechanisms for effective intervention, physiological tests often are not translatable to a clinically measurable outcome with which the patient can identify. Until sensitive and specific biomarkers are available to measure progression, a composite panel of clinical assessments is most appropriate.

Clinical assessments are recommended by the Movement Disorder Society (MDS) and a standard set of outcome measures recommended by the International Consortium for Health Outcomes Measurement (27). The MDS Unified Parkinson's Disease Risk Score [MDS-UPDRS; (28)] is the standard clinical measure for PD diagnosis and progression, though limited in detection of subtle improvements and susceptible to dopaminergic treatment effects and assessor subjectivity. Together, clinical measures of motor and non-motor symptoms, and quality of life, provide outcomes with relevance to the patient. These may be complemented with wearable devices and smart-phone applications that monitor PD specific behaviors (29). These technologies have the capacity to objectively measure changes in behaviors, including detailed information about patterns of movement, sleep quality, and blood pressure, with potential to develop computer programs to predict early indicators of PD, disease progression, and response to treatment. Determining which measures to assess requires consideration of data reliability and patient burden.

## Summary

Lifestyle has an important impact on risk and secondary prevention of many chronic conditions. There is increasing interest in the collection of lifestyle variables in PD cohorts. However, inadequate and lengthy self-reported recall surveys, the unlikelihood of lifestyle to have short-term or disease-modifying effects, and absence of objective outcome measures, are deterrents to capturing these data.

Given the complexity of symptoms in PD, the most viable therapeutic approach of lifestyle management may be multimodal. A combination of cognitive training, exercise, stress reduction, nutrition, and social components may be beneficial to quality of life. Whether these have a clinically significant effect on more objective health outcomes is best initially evaluated through longitudinal observational studies.

While there is much evidence on the benefits of lifestyle on general health outcomes (9, 10), such advice for people with PD must await a more concerted research effort to identify risk factors for disease progression. Then, implementation will require positive health promotion by health professionals, government, media, and policy makers. Health promotion initiatives can include prescribed exercise regimes, nutritional labels on foods, responsible marketing of tobacco and alcohol, and prioritizing wellbeing in educational and workplace organizations. While inducing long-term behavioral change is obviously a challenge, currently there is insufficient evidence to embark on such public health approaches in PD for most lifestyle factors, with the exception of exercise.

To enable a true overview of patient health and expedite research answers, data sharing and contribution to registries should be encouraged, and governments should prioritize resources for electronic data linkage between health services and research centers. The discovery of an evidence base around potential lifestyle modification in secondary prevention of PD progression depends on a much more robust and coordinated research effort world-wide than we have seen to date.

Modification of lifestyle risk factors is a foundational approach to prevention and management of chronic disease. These low-risk, self-managed therapies can empower the patient and reduce disease burden. Despite a robust evidence base in neurological diseases like stroke (10), there has been little coordinated effort to discover such evidence in PD. Considering the growing burden of PD, this is an important omission in modern PD research and needs to be addressed.

## Author Contributions

NN: conception, organization, execution, writing, editing and final approval of the manuscript, accountability for the work. GJ: conception, critical manuscript revision and final approval, accountability for the work.

### Conflict of Interest Statement

NN philanthropic funded fellowship, anonymous donor. GJ royalties for the books Overcoming Multiple Sclerosis and Recovering from Multiple Sclerosis.
